# Electronic Feedback in College Student Drinking Prevention and Intervention

**DOI:** 10.35946/arcr.v36.1.06

**Published:** 2014

**Authors:** Jessica M. Cronce, Joyce N. Bittinger, Junny Liu, Jason R. Kilmer

**Affiliations:** Jessica M. Cronce, Ph.D., and Jason R. Kilmer, Ph.D., are assistant professors; Joyce N. Bittinger, Ph.D., is a postdoctoral fellow; and Junny Liu is a postbaccalaurate research assistant in the Department of Psychiatry and Behavioral Sciences, University of Washington, Seattle, Washington. Dr. Kilmer also is the assistant director of Health & Wellness in the Division of Student Life at the University of Washington, Seattle, Washington.

**Keywords:** Alcohol use, abuse, and dependence, alcohol consumption, alcohol use associated effects and consequences, problematic alcohol use, risky drinking, intervention, prevention, college students, undergraduate student, college freshman year, technology, electronic health technology, Internet, World Wide Web, brief intervention, personalized feedback intervention (PFI), personalized normative feedback (PNF), randomized controlled trial, literature search

## Abstract

Alcohol consumption is prevalent among college students and can be associated with serious negative consequences. Several efficacious programs using one-on-one brief intervention techniques have been developed to target high-risk drinking by individual students, such as the Brief Alcohol Screening and Intervention for College Students (BASICS) (Dimeff et al. 1999). To reach a larger population (e.g., the incoming freshman class), researchers have adapted these interventions so that students can access them via the Internet or in some other electronic format. The purpose of this review is to discuss specific alcohol intervention programs that were (1) designed to be delivered remotely (e.g., via the Web or on an electronic device) without interaction with a provider and (2) were tested among college students using a randomized controlled trial design. Specific studies were drawn from earlier reviews as well as a comprehensive literature search. Although many programs have limited research support, and some findings are mixed, components that were directly translated from in-person BASICS to remote-delivery mediums (i.e., personalized feedback interventions [PFIs], personalized normative feedback [PNF] interventions), and broader programs that incorporate PFI/ PNF, show promise in reducing alcohol use and/or negative consequences. However, more research is needed and suggestions for how the field can move these interventions forward are discussed.

Compared with young adults not in college, college students exhibit higher rates of both regular alcohol consumption (67.7 percent vs. 53.9 percent) and heavy episodic consumption^1^ (37.4 percent vs. 29.5 percent) (Johnston et al. 2013) and are therefore at elevated risk for the myriad, and often costly, consequences related to alcohol misuse (Hingson et al. 2009; Perkins 2002). A variety of approaches to curtail high-risk drinking have been implemented over the years, including interventions aimed at the drinking behavior of individual students.

There has been a notable progression in individual-focused prevention efforts from purely educational programs, which typically emphasized potential life-altering consequences (e.g., grave injury, death) toward those that use alcohol-focused education to support alcohol skill use (e.g., refusal skills, protective behavioral strategies), placing primary focus on enhancing motivation and self-efficacy to act responsibly with respect to alcohol. The prototype for this latter approach is the Brief Alcohol Screening and Intervention for College Students (BASICS) (Dimeff et al. 1999), a brief motivational intervention (BMI) led by a facilitator trained in motivational interviewing (MI) (Miller and Rollnick 2013). In BASICS, each student participates in a one-on-one session to discuss personalized feedback related to alcohol use (i.e., the facilitator guides a discussion of the student’s alcohol use and consequences, their normative perceptions of other students’ drinking, their expectations about alcohol’s effects, etc., which were assessed prior to the session and are summarized for the student on a printed feedback sheet), coupled with education and skills training. Although the shift toward programs such as BASICS predates the 2002 report from the National Institute on Alcohol Abuse and Alcoholism’s (NIAAA’s) Task Force on College Drinking (NIAAA 2002), the compelling evidence for skills-based, motivational enhancement approaches highlighted in the Task Force report spurred the field to generate new interventions based on components of efficacious in-person programs, such as BASICS, that could reach a larger segment of the student body.

The first step toward bringing a BASICS-style BMI to a larger population was to test the effects of written personalized feedback delivered on its own, without a facilitator trained in MI (i.e., participants would receive feedback via U.S. mail) (e.g., Agostinelli et al. 1995; Larimer et al., 2007). With this approach, the written feedback was expanded to incorporate narrative explanations and supplemental material to replicate the information previously provided verbally by a trained facilitator. The approach has since been adapted for delivery via the Web, which has lower environmental and financial costs than the U.S. mail (i.e., no paper/envelopes, postage) and has become yet more attractive as technology (e.g., smartphones, tablets) evolved into the primary means by which young adults engage with the world and receive information.

Electronic alcohol feedback prevention programs (i.e., those designed to be delivered remotely, using some form of technology, typically the Web) include personalized feedback interventions (PFIs) that deliver most or all of the components included in the original BASICS feedback as well as personalized normative feedback (PNF) interventions that only deliver the normative re-education component of the BASICS feedback (i.e., educating participants about drinking norms and commonly held misperceptions about alcohol use among their peers). These programs are now common and include commercial and noncommercial branded interventions and interventions that are not branded, per se; the specifics of which may be unique to a single or small series of outcome studies. Some of these programs originally were designed to be focused on education but have since been modified (e.g., increasing focus on personalized feedback). Additional programs include some level of personalized feedback but do not rise to the level of what would constitute a PFI or PNF intervention. Given the range of available programs, this article will review the extant outcome literature for alcohol-specific, individual-focused, intervention programs designed for electronic delivery that include some level of personalized feedback, most of which may be considered a PFI or PNF intervention, that have been the subject of peer-reviewed, randomized controlled trials (RCTs) among college student populations.

The articles reviewed below were drawn from prior comprehensive qualitative reviews conducted by Cronce and colleagues (Cronce and Larimer 2011; Larimer and Cronce 2002, 2007), covering the span from 1984 to 2010, supplemented by a literature search of PsycInfo and Medline using comparable search terms with the stipulation that interventions be electronic (Web-based or delivered via an electronic device) and designed for administration outside of a controlled setting (although not always tested remotely). This strategy identified 29 new studies that utilized an RCT design and tested an electronic intervention for alcohol use within a sample of college students, reporting effects on one or more behavioral alcohol outcomes. These 29 studies are summarized in the table. Nearly all interventions were designed for delivery via the Web on a computer; therefore, unless otherwise stated, the reader should assume this is the method of intervention delivery. Effects on nonbehavioral outcomes, effects on use or consequences related to other drugs, comprehensive information on moderators and mediators of treatment effect, and full discussion of individual study limitations were considered beyond the scope of this review. Readers are referred to the original articles for more detailed information about a given study.

## Branded Programs That Include PFI-Style Information

### AlcoholEdu for College

AlcoholEdu for College incorporates personalized feedback regarding normative misperceptions and alcohol consumption, supplemented by education and skills training. Three studies reviewed by Cronce and Larimer (2011) (i.e., Croom et al. 2009; Hustad et al. 2010; Lovecchio et al. 2010) evaluated various versions of AlcoholEdu for College. Two additional publications reported on the effects of the intervention on alcohol use and consequences from a single multicampus study (Paschall et al. 2011*a*,*b*). Studies generally show reduced alcohol consumption and/or consequences (Hustad et al. 2010; Lovecchio et al. 2010; Paschall et al. 2011*a*,*b*) or a protective effect against increased alcohol use relative to assessment only (Lovecchio et al. 2010), at least in the short term (approximately 1 month). The largest study to date (Paschall et al. 2011*a*,*b*) utilized an intent-totreat, campus-wide implementation strategy and randomly assigned 30 campuses to either an intervention or control group. Treatment effects were observed in the fall semester (following implementation in summer and early fall) that were no longer evident by spring. Although stronger effects were found among campuses with higher rates of intervention participation, the lack of endurance of effects requires further research, perhaps using a longitudinal versus panel design. Studies are not universally positive, however. Croom and colleagues (2009) found that AlcoholEdu participants reported less participation in drinking games but no changes in consumption or consequences.

### AlcoholEdu for Sanctions

Whereas AlcoholEdu for College is advertised as a population-level prevention program for use with freshmen or the entire student body, AlcoholEdu for Sanctions specifically targets students who have been mandated to receive an alcohol intervention following a campus alcohol policy violation. The overall content of the program is similar to the original but emphasizes the prevention of future consequences and policy violations. One study reviewed by Cronce and colleagues (2011) (Carey et al. 2011) compared AlcoholEdu for Sanctions with a waitlist control group and at the 1-month followup found reductions in alcohol use, relative to the control group, for men only. Within-person reductions in alcohol use were reported in women in the intervention group, but no differences were found between women in the intervention and control groups. Likewise, within-person reductions in alcohol consequences were evident for men and women, but these reductions did not differ relative to the control group. No additional studies were identified, indicating a need for more research to establish efficacy.

### Check Your Drinking (CYD)

All iterations of CYD have included a brief online assessment followed by presentation of personalized feedback. Two studies reviewed by Cronce and Larimer (2011) (Doumas and Haustveit 2008; Doumas et al. 2009) evaluated the efficacy of the original beta version of CYD, showing reductions in both alcohol consumption among mandated students and high-risk-drinking intercollegiate athletes at the 1-and 3-month followup, respectively. Although the original beta version still is available, the program now is in its third iteration (version 3.0). Whereas studies have been conducted in the general adult population, to date, CYD 3.0 does not seem to have been specifically evaluated among college stu-dents. Therefore, research is needed to establish the efficacy of the most current iteration in college populations.

### College Drinker’s Check-up (CDCU)

CDCU is a Web-based adaptation for college students of the well-established in-person intervention known as the Drinker’s Check-up, originally developed for heavy-drinking adults. Like its predecessor, the CDCU begins with a screening instrument and incorporates decisional balance exercises (i.e., assessing and considering pros and cons of drinking) along with personalized feedback. A single two-trial study (Hester et al. 2012) has evaluated CDCU. In the first trial, reductions in peak blood alcohol concentration (BAC) were significant (correcting for multiple comparisons) at 1 month compared with repeated assessment but were absent at 12 months. The second trial, comparing CDCU to postassessment only (versus repeated assessment) found robust reductions across peak and typical drinking outcomes from baseline to 1 month. Although preliminary evidence suggests that this program may be efficacious, limited evidence, in addition to the sole finding of reduced peak BAC compared with repeated assessment, points to the need for further evaluation before the program should be widely adopted.

### e-CheckUpToGo

E-CheckUpToGo, called e-CHUG in earlier versions, incorporates assessment, personalized feedback targeting normative misperceptions and other alcohol behaviors, education, and skills training. Three of the four previously reviewed studies on this approach demonstrated at least short-term positive effects on alcohol use (Doumas and Andersen 2009; Hustad et al. 2010; Walters et al. 2007) and alcohol-related consequences (Doumas and Andersen 2009). Five new studies have been published since the 2011 review by Cronce and Larimer, three of which show reductions in various indices of alcohol use (Doumas et al. 2010, 2011*a*) and/or consequences (Alfonso et al. 2013) relative to control subjects across follow-up periods ranging from 1 to 6 months. One study (Murphy et al. 2010) demonstrated no between-group differences at 1 month compared with assessment only, although the study did show with-in-group differences for e-CheckUpToGo. Another study showed successes compared with an in-person BMI at 1 month that were no longer present at the 8-month followup, with increased drinking evident in the e-CheckUpToGo group (Doumas et al. 2011*b*,*c*). Absence of an assessment-only control in this study leaves overall efficacy unclear. Although e-CheckUpToGo has been tested across an array of high-risk populations (e.g., mandated students, athletes, and freshmen), research on any one college population is relatively limited and would benefit from replication, especially given variation in specific effects on alcohol outcomes across studies.

### MyStudentBody (MSB) and MyStudentBody-Parent(MSB-P)

MSB includes general education and skills training, along with assessment and personalized feedback discussing alcohol behavior, beliefs, and risks. One previously reviewed study (Chiauzzi et al. 2005) evaluated MSB among binge-drinking college students. Participants randomly assigned to MSB showed reductions in peak drinks per drinking day and composite drinking index scores at 1 month but were no different than an alcohol education control group at 3 months. Female, but not male, MSB participants showed reduced consumption on special occasions and fewer alcohol-related negative consequences relative to control subjects at followup. Additional research is needed to evaluate efficacy.

More recently, Donovan and colleagues (2012) examined MSB-P, a modification of MSB delivered to parents (only) that encourages parent–teen communications about alcohol. Parent–teen dyads were randomly assigned to either MSB-P or an attention control (i.e., receipt of an equal amount of material that is not expected to produce change, in this case, an alcohol education e-mail newsletter). Parents received the intervention 4 weeks prior to the start of their child’s freshman year of college. Assessments through 6 months postintervention found no impact on students’ binge drinking, which was the single alcohol use outcome variable.

### Unitcheck

Unitcheck provides personalized feedback on alcohol consumption as well as related education and advice. One previously reviewed study (Bewick et al. 2008) demonstrated that drinks per drinking occasion were reduced at 12 weeks postintervention compared with assessment only. Subsequently, Bewick and colleagues (2010) randomly assigned students reporting alcohol use in the past 6 months to immediate access to the intervention (weeks 1 to 7), delayed access (weeks 8 to 15), or assessment only. Results were mixed. Reductions in drinks per drinking occasion occurred for the delayed and assessment-only conditions but not in the immediate condition. Across conditions, participants who completed a minimum of two of five assessments reduced drinking with additional reduction for those assigned to the intervention arms. This study demonstrated that repeated assessment alone may be effective at reducing alcohol consumption, and this may be enhanced by participation in an intervention such as Unitcheck. As with many programs, more research is needed.

## Unbranded PFI-style and Personalized Normative Feedback (PNF) Programs

A number of studies have examined the effects of unbranded PFIs and/or single-component PNF interventions, the features of which differ, and any one version may only be represented by a single study. Two previously reviewed studies evaluated unbranded electronic PFIs with generally positive findings. Compared with a control group, Kypri and colleagues (2004) showed reduction of alcohol use and consequences, and, comparing a minimal versus enhanced version of PFI, Saitz and colleagues (2007) found within-person reductions in alcohol use and problem severity among women and in problem severity, but not consumption, among men across active interventions. Evaluating a brief computer-based PNF, Neighbors and colleagues (2004) found reductions in drinking persisting up to 6 months.

Twelve subsequent studies have tested other unbranded PFIs or PNFs. Similar to Saitz and colleagues (2007), Kypri and colleagues (2008) compared two versions of a PFI (a single vs. multiple dose) but also included an education-only control condition. Students scoring 8 or more on a 10-question screening instrument (i.e., Alcohol Use Disorders Identification Test [AUDIT]) were recruited from primary care. Relative to a control group, a single dose of a PFI resulted in lower frequency of drinking at 6-month followup, lower total consumption and academic consequences at both 6-and 12-month followup and reduced alcohol problems at 12 months. The multidose condition resulted in decreased typical quantity and frequency of drinking, lower total consumption, and reduced frequency of heavy episodic drinking at the 6-month followup; reduced academic consequences at both the 6-and 12-month followup, and reduced non-academic consequences and alcohol problems at the 12-month followup.

Kypri and colleagues (2009) compared a two-dose PFI to assessment-only among Australian college students who scored 8 or more on the AUDIT and engaged in at least one occasion of heavy episodic consumption over the previous 4 weeks. Participants received assessment and feedback at baseline and again 1 month later, including additional feedback on alcohol use and consequences that occurred after the initial feedback. Of outcomes examined at 1-month followup, participants receiving the two-dose PFI reported a lower frequency of drinking, fewer drinks per occasion, and lower total consumption relative to those who received assessment only. Only the effects on frequency of drinking and total consumption were maintained at the 6-month followup. Negative-consequence variables did not differ at either time point. Overall differences in alcohol consumption differed by condition, with the intervention group consuming 17 percent less alcohol compared with an 11 percent reduction by the control condition. The authors indicated that this was primarily driven by reductions in frequency of drinking rather than amount consumed per episode.

Kypri and colleagues (2014) compared a PFI to assessment only among students scoring 4 or more on the AUDIT-C^2^ at seven New Zealand universities. At the 5-month followup, those randomly assigned to PFI reported fewer drinks per typical drinking occasion; however, this effect was reduced to non-significance in sensitivity analyses designed to detect effects of differential attrition. No effects on the five other drinking-related outcomes assessed were evident.

Palfai and colleagues (2011) randomly assigned college students scoring 8 or more on the AUDIT to PFI versus attention control. At the 1-month followup, participants who received the PFI reported drinking fewer drinks per week overall. Subsequent analyses indicated that this effect was driven by those students who had reported a greater number of alcohol consequences at baseline, with no effect of the intervention among students with a lower number of baseline consequences. A similar effect was shown for heavy episodic consumption, with reductions in episode frequency evident among those with greater baseline consequences and not for those with fewer baseline consequences.

Martens and colleagues (2010) compared two forms of PFI—one targeted to college athletes and the other aimed at college students in general (generic)—against an alcohol education control group among varsity and club-sport athletes. At 6 months, those in the targeted PFI condition reported lower peak BAC compared with the control group and the generic PFI, with increases in peak BAC evident in these latter two groups. However, for heavy drinkers, reductions in peak BAC were evident for both PFI conditions compared with the control group. No effects were found for other alcohol-related indices.

Bryant and colleagues (2013) randomly assigned students to receive either a PFI or educational information on the risks of alcohol via e-mail. Followup at 6 weeks postintervention revealed that those who had received the PFI reported fewer drinks per week and fewer days drunk in the past 30 days compared with those who received education only. However, it must be noted that about 40 percent of students were lost to followup, and these individuals reported significantly higher values on all alcohol outcome measures at baseline.

LaBrie and colleagues (2013) compared a full PFI to eight versions of a PNF intervention (a component of the full PFI) that varied the specificity of the normative reference group and a generic non–alcohol-focused normative feedback control group in Caucasian and Asian students reporting one or more occasions of heavy episodic consumption in the past month. PFI participants reported lower peak drinking and fewer drinking days compared with control subjects, with no effects on alcohol consequences. Those receiving any PNF reported lower average total consumption, lower peak drinking, fewer drinking days, and fewer alcohol consequences compared with control subjects. Comparisons of PNF conditions indicated that use of the “typical student” reference group is most effective.

Lewis and colleagues (2014) expanded targets of PNF to include alcohol-related risky sexual behaviors (RSB) in addition to alcohol-related behaviors. Students were stratified by gender and level of drinking and randomly assigned to an alcohol-only PNF, an alcohol-related RSB-only PNF, a combined alcohol and alcohol-related RSB PNF, or assessment only. The alcohol-only and the alcohol-related RSB-only PNFs each reduced their target behaviors and the combined intervention reduced both sets of outcomes relative to control subjects. None of the interventions reduced alcohol-related consequences. Results indicate that combining related treatment targets may be an effective strategy.

Ekman and colleagues (2011) compared a minimal feedback intervention, in which participants’ own drinking was compared with safe-drinking guidelines, to PNF with harm reduction advice among students at a Swedish university. Retention rates at the 3-and 6-month followup were quite low (between 24 percent and 38 percent), and although some significant within-person reductions in alcohol use and risk were evident, given the small sample size, it was not surprising that no significant between-groups effects emerged.

Moreira and colleagues (2012) evaluated PNF against assessment-only and delayed (posttest–only) assessment in a sample of students drawn from multiple universities in the United Kingdom. Although retention was poor (50 percent) at the 6-month followup, a significant decrease in weekly drinking was evident in the PNF group compared with control subjects. However, this effect was absent at the 12-month followup, and no effects were observed on any of the other alcohol outcome measures.

Neighbors and colleagues (2010) tested gender-specific versus non–gender-specific PNF as a single-versus four-dose (biannual) intervention against an attention control group among heavy-drinking freshmen. At 6 months, those in the four-dose, gender-specific PNF condition reported lower weekly drinking compared with the control group. Women, but not men, who received the four-dose, gender-specific PNF decreased their alcohol problems compared with control subjects. No differences were found on any outcome between the control group and the gender-specific single-dose PNF or non–gender-specific PNF groups.

Finally, Mason and colleagues (2014) randomly assigned students with hazardous drinking to either an assessment-only control condition or a very brief (four to six texts over 4 consecutive days) automated text intervention including personalized information on drinking frequency, social norms, social risk, and protective behavioral “boosts,” if requested. The amount of personalized information contained in the intervention is most consistent with a PNF versus a PFI; however, the inclusion of skills training and the MI framework used for the texts go beyond a standard PNF. This was a small-scale proof-of-concept investigation to determine feasibility. Although there were no significant group differences on behavioral alcohol outcomes, this was not surprising given the very small sample size. The results did show changes in potential mediators of intervention efficacy (i.e., readiness to change), suggesting further research may be warranted.

## Event-Specific Prevention (ESP)

Electronic interventions targeting general alcohol misuse have been adapted to proactively address alcohol use and consequences for specific events associated with extreme alcohol consumption (i.e., 21st birthdays, spring break [SB]). In an ESP study reviewed by Cronce and Larimer (2011), Neighbors and colleagues (2009) randomly assigned participants to receive an electronic card 2 days before their 21st birthday that contained a hyperlink to personalized feedback about their drinking intentions and anticipated BAC for their 21st birthday, associated normative information, education on BAC effects, and suggestions for protective behavioral strategies. The intervention (which is most consistent with PNF) reduced reported BAC levels on the day of participants’ 21st birthdays compared with an assessment-only control condition. This effect was pronounced for those with baseline intentions to reach higher BACs.

Three subsequent ESP studies were identified. In the first, Neighbors and colleagues (2012) tested a 21st birthday– specific in-person BASICS, a Web-based 21st birthday PFI, a general in-person BASICS condition, and attention control. Two additional conditions tested augmented versions of the 21st birthday–specific interventions by incorporating a friend of the participant who was supplied with alcohol education and harm reduction tips for their friend’s birthday celebration. Students with reported intention to “binge drink” on their upcoming 21st birthday were randomly assigned to one of the six conditions. Results were mixed. None of the interventions reduced the number of drinks consumed compared with the control group. The 21st birthday PFI without the friend component, but not with, resulted in lower BACs compared with control subjects, as did the general in-person BASICS. Unlike the 21st birthday PFI without the friend component, the 21st birthday PFI with the friend component reduced consequences relative to the control group, as did all three in-person conditions.

With a similar design to Neighbors and colleagues (2012), Lee and colleagues (2014) conducted a large RCT examining five different intervention conditions against an attention control with the goal of reducing drinking and negative drinking consequences over SB. Two of five interventions included a PFI that was designed specifically to address SB drinking; one with a friend component, one without. Neither SB-PFI, with or without a friend, nor the original in-person BASICS, was shown to be effective in reducing SB drinking. Only the in-person SB-BASICS intervention without a friend reduced drinking compared with control subjects. Of note, the same intervention with the friend component was not effective.

Lastly, Patrick and colleagues (2014) applied a PFI modified to address both alcohol-related behavior and alcohol-related RSB, similar to Lewis and colleagues (2014), as an ESP to target SB alcohol use. Students between the ages of 18 and 21 who planned to go on SB trips with friends were randomly assigned to PFI or assessment only. Although normative perceptions were reduced, there were no main effects on any of the primary alcohol-related behavioral outcomes.

## Other Programs with Minimal Personalization

In addition to unbranded PFIs, other interventions have taken advantage of technology-based delivery methods that include some personalization but which cannot be considered a full PFI or PNF intervention. For example, Cronce and Larimer (2011) reviewed a study by Weitzel and colleagues (2007) that compared 2 weeks of repeated (daily) assessment on a handheld (HH) computer plus tailored feedback on avoiding alcohol consequences, based on baseline levels of reported self-efficacy and drinking outcome expectancies, to repeated assessment alone. Those who received the tailored feedback messages reported fewer drinks per drinking day on the HH device during the daily assessment period. However, no group differences in drinking outcomes were evident on the retrospective assessment for the same period completed at the 2-week followup.

Hendershot and colleagues (2010) tested an intervention that targeted the ALDH_2_ genotype, found almost exclusively in individuals of northeast Asian descent, which can convey a protective effect against alcohol misuse. Students of 100 percent Chinese, Korean, or Japanese heritage underwent genotyping and were randomly assigned to personalized genetic feedback that included their ALDH_2_ test results and information specific to their genotype (ALDH_2_ 1/1, ALDH_2_ 1/2, ALDH_2_ 2/2), or attention-control feedback that provided normative information about nonalcohol behaviors. At the 1-month followup, only the group with one of two affected alleles (ALDH_2_ 1/2) demonstrated a reduction in alcohol-related measures (i.e., peak quantity, typical weekend quantity, drinking frequency). However, this is an encouraging result as this genotype is most at risk for alcohol-related cancers.

Schuckit and colleagues (2012) examined a prevention paradigm based on another genetically linked trait, subjective levels of response (LR) to alcohol (high vs. low). Freshman were randomly assigned to either (1) a low LR–based prevention group (LRB group), which watched four 45-minute Internet-based videos that included, in addition to prevention messages, information on how low LR to alcohol may promote heavy drinking; or (2) a non-LRB comparison group, which saw the same prevention messages without the LR framework. Self-reported usual and maximum drinks per drinking occasion decreased significantly for all participants regardless of LR status or condition. Low-LR students showed the greatest decreases in the LRB condition and high-LR students showed greater decreases in the non-LRB condition, demonstrating support for tailoring prevention messages to specific predisposing factors such as LR. Because the study design did not include an assessment-only control group, general efficacy information is unknown.

Hagger and colleagues (2012) randomly assigned students from the United Kingdom to one of four instruction conditions delivered using Web and e-mail: implementation intention only (setting specific intentions to reduce alcohol intake), mental simulation only (visualizing achieving goals), a combination of the two, and an assessment-only control. Only the students in the mental simulation–only condition reduced alcohol consumption and heavy episodic drinking occasions over the subsequent month compared with the control group. Students with the highest baseline use, however, had a greater reduction in alcohol consumption in the combined condition than any of the other conditions.

### Alcohol 101+

Alcohol 101+, a Web-based modification of the earlier CD-ROM–based Alcohol 101 program, provides alcohol education and skills training using a “virtual campus,” modeling potential drinking situations and discussing possible consequences and alternatives, with personalized BAC calculations provided. Three studies were identified, two of which (Carey et al. 2009, 2013) included Alcohol 101+ as a control condition, limiting the ability to evaluate efficacy. The third (Carey et al. 2011), previously reviewed by Cronce and Larimer (2011), compared Alcohol 101+ with a waitlist control group and found reductions in alcohol use for male mandated students compared with wait-listed students at 1 month. However, only within-person reductions (no between-groups effects) were found for female mandated students. In terms of alcohol consequences, women assigned to Alcohol 101+ actually fared worse compared with waitlist students, and there were no intervention effects for men at 1 month.

### Michigan Prevention and Alcohol Safety for Students(M-PASS)

M-PASS comprises 4 10-to 15-minute online MI sessions delivered over 9 weeks. Sessions were tailored based on the participants’ general drinking profiles, readiness to change and self-efficacy, and included some personalized information (i.e., drinking norms based on participant’s demographics). One study has evaluated the efficacy of the M-PASS program, with findings from posttest (Bingham et al. 2010) and 3-month followup (Bingham et al. 2011) published separately. Treatment effects, relative to the control group, varied somewhat by gender, with lower binge drinking frequency among high-risk drinking men, fewer total drinks consumed over the past 28 days among high-risk drinking women, and fewer drinks per drinking day among low-risk drinking women at posttest. At 3 months, male high-risk participants in M-PASS continued to show lower frequency of heavy episodic consumption compared with control subjects; however, the effect would not have been significant if a correction for multiple comparisons was applied. Treatment effects for women differed at the 3-month followup relative to posttest, with lower frequency of heavy episodic consumption and fewer alcohol related consequences among high-risk women relative to control subjects. The availability of a single study and the variability of findings over time indicate that additional research is needed before strong conclusions regarding efficacy can be drawn.

## Discussion

College student alcohol use remains a critical issue. Fortunately, there have been successful advances in prevention strategies targeting individuals to reduce the harms associated with college student drinking. It is important to stress that no one program or approach is sufficient to prevent or reduce high-risk drinking, and an overall strategic plan should incorporate multiple approaches targeting every level of intervention (i.e., universal, selective, and indicated). Whereas the amount and quality of research on any one program varies, the extant evidence suggests that electronic interventions may be one piece of an effective overall strategic plan.

Although the general PFI approach (grouping together commercially branded and unbranded programs) and PNF approach seem to be efficacious on the whole, data are insufficient to make general recommendations regarding the best program for adoption. Moreover, overall conclusions regarding the efficacy of electronic interventions globally, and any one program, must be tempered by the limitations of the individual studies (e.g., small sample sizes, poor retention) as well as the challenges and limitations imposed by rapidly changing technology (e.g., devices and Web browsers are not universal, requiring unique adaptations of interventions; innovations make hardware outdated within 1 year) and specifics of the campus environment and resources (e.g., availability of programming staff to monitor compliance; ability to impose contingencies on students who do not complete the intervention, such as holding grades or preventing registration). Certainly, additional research is needed, and efforts to replicate existing findings are indicated. Of note, many of the programs reviewed have been subject to modifications over time, resulting in multiple iterations or versions. Colleges wishing to implement one of these programs should conduct due diligence before adoption to understand which variant they are considering and to determine the empirical support for that specific version, as efficacy research on one version may not apply to others. For commercially available programs, colleges can, and should, request articles supporting efficacy for the current version that would be adopted on their campus to evaluate the potential benefit of implementation.

In addition to program choice, campuses may wish to consider for whom such approaches should be made available (e.g., first-year students, athletes, Greek members, mandated students, etc., which can be informed by research efforts to determine for whom these approaches are most helpful) and must also critically consider potential limitations of electronic interventions. For example, research has shown that without incentives or penalties for noncompliance, students are unlikely to complete interventions of their own volition (see Paschall et al. 2011*a*,*b*). Likewise, without face-to-face interaction with a person who can assess and confirm the degree to which a student is paying attention (as would be the case in an intervention like BASICS), a potential limitation includes the degree to which students are engaged in, connected to, and even multi-tasking during the intervention. Additionally, given the high variability in length and content across different electronic interventions, the appropriate intervention “dose” given to any individual student to decrease his or her alcohol use (and the consequences he or she has experienced) needs to be more firmly established (as does the need for any “booster” sessions beyond the initial intervention to potentiate and/or sustain effects). Although the effect of electronic interventions on alcohol-related negative consequences does not seem to be as robust as in-person BMIs (as they are only evident in a minority of the studies detailed here), followup generally has been shorter in studies of PFIs and PNF interventions relative to BMIs and it may be that longer follow-ups are needed to demonstrate an effect on consequences. Other factors also may be at work, such as differences across studies in assessment tools used to measure consequences. Thus, more research is needed to specifically address under what conditions electronic interventions produce reductions in negative consequences.

In terms of future research, there are several interesting and important questions that need to be addressed in order to maximize the potential of electronic interventions. Briefly, these include the study of:

- *Additional interventions. Other available programs would benefit from more thorough empirical validation, such* as Alcohol-Wise, an educational program that contains e-CheckUpToGo, or MyPlaybook, a program targeted toward athletes. Although preliminary findings have been presented at informal academic venues, no peer-reviewed published RCTs were identified for these programs.
- *Timing of the intervention.* Many campuses require first-year students to complete an alcohol intervention prior to matriculation. Although this may convey the seriousness with which a campus takes alcohol prevention and serve to get students on the “same page” regarding alcohol information, students may not yet have a sense of general college norms, what goes on at their school, or what pressure to drink is like. Research could explore what, if any, boosters might be needed once students arrive on campus and if there is an optimal time for intervention delivery.
- *Opportunities for reaching more advanced students.* Given the emphasis on entering/first-year students, how might electronic interventions systematically be offered to students in later years of study? For example, research by Neighbors and colleagues (2009, 2012) suggests that students turning 21 could be invited to participate in an ESP. However, when not required (as with entering students), how might we attract students to participate in such interventions?
- *Electronic PFIs as a referral option.* Alcohol screening in campus health and counseling centers helps identify students struggling with substance use and reduce the likelihood of students “slipping through the cracks.” Hingson (2010) suggested that if schools implement such screenings, there would be an impact at the campus level through referral to empirically supported interventions. As primary care–based BMIs typically are in person, determining what circumstances and for whom referral to an electronic PFI (adjunct or standalone) would be effective should be examined.
- *Keeping abstainers in mind.* Studies have shown a protective effect of personalized feedback for those who do not drink. For example, in a mailed feedback intervention, Larimer and colleagues (2007) demonstrated that abstainers who received the feedback were twice as likely to be abstaining 1 year later compared with control participants. With increased risk for addiction associated with earlier onset of use, delaying the initiation of use can be of great public health importance. The role of electronic interventions in achieving this goal should be explored and abstainers considered as schools develop a strategic plan.
- *Duration/length and formatting of interventions.* How brief can a brief intervention be and still be effective? Without a facilitator present, how much information is necessary to have an impact? In addition, as more online information is viewed on smaller tablets and phones, the ability to impact change in a time-and space-efficient way will increase in importance.

## Conclusion

As reviewed here, the existing evidence gives us reason to be excited about the potential of electronic feedback interventions in reducing high-risk drinking and related harm among college students. That said, the field is still young and research must be done to establish the parameters of successful intervention, as well as the reliability, relative efficacy, and longevity of effects related to specific electronic programs. PFI-style programs have the most research support to date, but the increasing variety of style and content of PFIs, including among electronic programs with different iterations, makes it harder to group these programs together when discussing efficacy but also points to the potential for campuses to develop their own PFI based on features of programs with promising outcomes. Whereas this review summarizes the existing base of information on electronic alcohol feedback interventions, research is always advancing. Campuses wishing to adopt a given program are again advised to “do their homework” to ensure their expenditure of resources and dedication to one specific program is based on the most up-to-date and accurate information.

## Figures and Tables

**Table:** Summary of Methodologies and Outcomes for Previously Unreviewed Studies Included in the Current Review

Authors Year	Group Studied	Intervention Condition	Behavioral Alcohol Assessment/Outcome Measures	Follow-up Assessment	Conclusions/Results For Electronic Intervention Condition(s)
Alfonso et al. 2013	Undergraduate students who were mandated to an alcohol intervention for violating university alcohol policies (*N* = 173).	Brief Alcohol Screening and Intervention for College Students (BASICS) (individual in-person brief motivational intervention [BMI]); CHOICES (group in-person); e-CheckUpToGo (individual personalized feedback intervention [PFI]).	Alcohol Timeline Followback; BAC; Rutgers Alcohol Problem Index.	3 months	e-CheckUpToGo was associated with significant within-person reductions in alcohol-related harms, which were similar to those observed for the BASICS condition. No reductions were evident on indices of alcohol use for those receiving e-CheckUpToGo.
Bewick et al. 2010	University students (ages 18–67; 95 percent undergraduates) reporting consumption of alcohol at least once every 6 months (*N* = 1,112); 57 percent of the sample scored 8 or higher on the AUDIT.	Immediate (weeks 1 through 7) vs. delayed (weeks 8 through 15) access to the Unitcheck electronic intervention vs. assessment only control.	Retrospective weekly drinking diary, AUDIT.	4 follow-up assessments across the 24-week study	Significant reductions in drinks per drinking occasion were evident in the delayed intervention and assessment-only conditions, with no effect in the immediate intervention condition. Those assigned to either intervention condition that completed more than two of the five total assessments showed greater reductions in drinking than those in the control condition.
Bingham et al. 2010	Freshmen college students who were living in dormitory housing (*N* = 1,137); sample divided into non-, low-, and high-risk drinkers for analyses. High-risk defined as consumption of an average of more than 14 (male) or 7 (female) drinks per week or 5 (male) or 4 (female) drinks in a row at least 2 times during the past 3 months. Nondrinkers reported no alcohol consumption in the 6 months preceding baseline.	Four sessions of online *Michigan Prevention and Alcohol Safety for Students* (M-PASS) program vs. assessment-only control.	Daily drinking questionnaire, 28-day Timeline Followback (TLFB), Young Adult Alcohol Problems Screening Test (YAAPST).	Posttest at end of intervention (9 weeks)	Among those assigned to M-PASS relative to control: high-risk male drinkers reported fewer episodes of heavy drinking; high-risk female drinkers reported lower total drinks on TLFB; low-risk female drinkers report fewer drinks per drinking day.
Bingham et al. 2011	3-month followup of sample reported in Bingham et al. (2010).	See Bingham et al. (2010).	See Bingham et al. (2010)	3 months after intervention end	Among those assigned to M-PASS relative to control: male and female high-risk drinkers reported fewer episodes of heavy episodic consumption and high-risk female drinkers also reported fewer alcohol-related consequences. Further, M-PASS showed protective effect among nondrinking women in terms of total drinks consumed.
Bryant et al. 2013	Students enrolled in first-year psychology courses (*N* = 191).	E-mailed PFI vs. e-mailed educational information about the risks of alcohol consumption.	AUDIT, Daily Drinking Questionnaire, Rutgers Alcohol Problem Index.	6 weeks	Relative to alcohol education, e-mailed PFI was associated with fewer drinks per week and fewer days drunk in the past 30 days.
Carey et al. 2013	College students who were mandated to an alcohol intervention for first-time campus alcohol policy violations (*N* = 288).	BMI or Alcohol 101+ program: self-chosen (*N* = 147) vs. randomly assigned (*N* = 141).	Daily Drinking Questionnaire; AUDIT; Brief Young Adult Alcohol Consequences Questionnaire.	1 and 2 months	Reductions in alcohol use and consequences were evident among those receiving the BMI relative to Alcohol 101+ at the 2-month followup. The absolute efficacy of Alcohol 101+ cannot be determined due to the absence of an assessment control condition; however, those who were randomly assigned to Alcohol 101+ showed greater reductions in drinks per drinking day and drinks per week relative to those who chose Alcohol 101+.
Donovan et al. 2012	High-school seniors and their parents (*N* = 279 parent–teen pairs, of which *N* = 150 who reported drinking and were included in analyses regarding alcohol use).	MyStudentBody-Parent (MSB-P) online intervention vs. attention control (e-mailed alcohol education newsletters).	Single question assessing number of heavy-drinking episodes in the past 30 days using 5/4 gender-specific criteria within 2-hour time frame on a given occasion.	1 week postintervention, 3 and 6 months	No treatment effect on proportion of teens reporting episodes of heavy drinking.
Doumas et al. 2010	First-year NCAA Division 1 intercollegiate athletes (*N* = 106); sample divided into low- and high-risk drinkers for analyses. High-risk defined as reporting one or more occasions of heavy drinking in the past 3 months using the 5/4 gender-specific criteria.	e-CheckUpToGo vs. Web-based alcohol education program.	Daily Drinking Questionnaire.	3 months	Relative to control, high-risk drinkers in the e-CheckUpToGo condition significantly reduced their weekly drinking, peak drinking quantity and frequency of drinking to intoxication. There were no differences among low-risk drinkers.
Doumas et al. 2011*a*	Freshmen college students randomly assigned as intact orientation groups (*N* = 82); sample divided into low- and high-risk drinkers for analyses. High-risk defined as reporting one or more occasions of heavy drinking in the past 3 months using the 5/4 gender-specific criteria.	e-CheckUpToGo vs. assessment-only control.	Daily Drinking Questionnaire; Rutgers Alcohol Problem Index; individual items assessing peak alcohol consumption and frequency of drinking to intoxication.	3 months	Relative to control, high-risk drinkers in the e-CheckUpToGo condition significantly reduced their peak drinking quantity and frequency of drinking to intoxication. However, only seven participants were in the high-risk e-CheckUpToGo condition.
Doumas et al. 2011*b*	Students mandated to university counseling services for violating university alcohol policies (*N* = 37).	Online e-CheckUpToGo feedback only (PFI) vs. counselor-facilitated review of e-CheckUpToGo feedback (BMI).	Daily Drinking Questionnaire; Rutgers Alcohol Problem Index; individual items assessing peak alcohol consumption and frequency of drinking to intoxication.	30 days	Participants in both conditions showed significant within-person reductions in weekly and peak drinking quantity, frequency of drinking to intoxication, and alcohol-related consequences. No significant differences were found between the groups.
Doumas et al. 2011*c*	8-month followup of sample reported in Doumas et al. (2011*b*) (*N* = 83).	See Doumas et al. (2011*b*).	See Doumas et al. (2011*b*).	8 months	Relative to those in the e-CheckUp-ToGo PFI condition, participants in the BMI condition showed significant reductions in weekly drinking quantity and frequency of heavy episodic drinking. Participants in the PFI condition showed increases on these drinking indices.
Ekman et al. 2011	Sophomore students from a single Swedish university who consumed 180/120 (men/women) grams of alcohol or more per week in the past 3 months and/ or consumed 60/48 (men/ women) grams of alcohol or more on two or more occasions in the past month (*N* = 158).	Personalized normative feedback (PNF) with harm reduction tips compared with a minimal feedback control (comparing the student’s drinking to national safe drinking guidelines).	Items assessing average weekly alcohol consumption, frequency of heavy episodic drinking and peak BAC; specific measures used were not indicated.	3 and 6 months	Significant within-person reductions in weekly consumption in the PNF group, and significant within-person reductions in number of heavy drinking episodes in both conditions at both followups. No significant between-group differences for alcohol-related outcomes at either time point.
Hagger et al. 2012	Undergraduate students from a single university in the United Kingdom (*N* = 238).	Web-based instructions: 2 (mental simulation of achieving goal of keeping drinking within safe limits vs. no mental simulation) × 2 (intention to implement reduction in drinking vs. no implementation intention) design.	Items assessing number of alcohol units consumed and number of episodes of heavy drinking in the past 4 weeks using criteria applicable in the United Kingdom; specific measures used were not indicated.	1 month	Receipt of the mental simulation instructions without the implementation intention instructions was associated with reductions in number of units consumed and heavy episodic drinking.
Hendershot et al. 2010	College students of northeast Asian descent (*N* = 200).	Web-based ALDH2 genotype-specific feedback (ALDH2*1/*1, ALDH2*1/*2, or ALDH2*2/*2) vs. attention control.	Daily Drinking Questionnaire.	30 days	Participants heterozygous for the ALDH 2*2 allele (i.e., ALDH2*1/*2) who received genetic-risk feedback personalized to their genotype showed reductions in quantity and frequency of drinking relative to control.
Hester et al. 2012	College students who reported one or more occasion of heavy episodic drinking in the past 2 weeks using the 5/4 gender-specific criteria with an associated blood alcohol content [BAC] of .08%. (Two trials: *N* = 130 and *N* = 81).	College Drinkers Check-up (CDCU). In experiment 1, CDCU vs. assessment-only control; in experiment 2: CDCU vs. a delayed-assessment control group.	AUDIT, Brief Drinker’s Profile, 19 items from the CORE Institute’s alcohol survey related to negative consequences.	Experiment 1: 1 and 12 months; Experiment 2: I month	Experiment 1: Adjusting for multiple comparisons, reductions in peak BAC on two heavier occasions in the past month were evident at 1-month followup among those assigned to CDCU, but the effect was absent at 12 months. Experiment 2: CDCU associated with significant reductions in drinks per week, typical peak BAC, and average number of drinks and BAC on two heavier occasions in the past month.
Kypri et al. 2008	Students at a New Zealand student health service scoring 8 or higher on the AUDIT (*N* = 429).	Single-dose PFI vs. two-dose PFI vs. education-only control.	AUDIT, additional items assessing frequency of drinking, typical quantity per occasion, total volume, frequency of heavy drinking episodes (120/80 g, men/women), consequences of heavy drinking; specific measures used were not indicated.	1, 6, and 12 months	Reductions in frequency of drinking, total consumption, and academic consequences at 6 months in both PFI conditions relative to control. Additional reductions in frequency of drinking, typical quantity, and frequency of heavy episodic consumption at 6 months in the multidose PFI condition. Reductions in total consumption and academic problems were still evident at 12 months in the single dose PFI condition. Reductions in academic problems were also still evident at 12 months in the multidose condition, and effects on nonacademic consequences emerged. Reductions in AUDIT scores (alcohol problems) were evident in both PFI groups at 12 months.
Kypri et al. 2009	Undergraduates at a single Australian university who scored 8 or higher on the AUDIT and who exceeded Australian gender-specific standards for one or more episodes of heavy episodic drinking in the past 4 weeks (*N* = 1,904 at 1-month followup; 1,578 at 6 months).	Two-dose PFI vs. assessment only control.	AUDIT, Alcohol Problems Scale (APS), Academic Role Expectation and Alcohol Scale (AREAS), additional items assessing frequency and quantity of drinking, and heavy-drinking episodes.	1 and 6 months	Relative to control, participants in the PFI condition reported significant reductions in frequency and quantity of drinking (drinks per occasion and total consumption) at 1-month followup; effects on frequency of drinking and total consumption were maintained at 6 months.
Kypri et al. 2014	Non-Maori students at seven New Zealand universities who scored 4 or higher on the AUDIT-C (*N* = 2,850).	PFI including screening for, and feedback regarding, alcohol dependence vs. assessment only.	AUDIT-C, AREAS, additional items assessing alcohol use; for intervention participants only: AUDIT, Leeds Dependence Questionnaire.	5 months	PFI with dependence screening and feedback resulted in fewer drinks per drinking occasion at followup; however, analyses accounting for attrition call this finding into question. No effects evident on five other indices of alcohol use.
LaBrie et al. 2013	Heavy-drinking Caucasian and Asian undergraduates at two West Coast universities (*N* = 1,663).	Web-based PFI vs. eight Web-based PNF conditions differing on level of specificity of student-normative referent groups: typical same-campus student or a same-campus student at one (either gender, race, or Greek affiliation), or a combination of two, or all three levels of specificity vs. non-alcohol normative feedback control.	Daily Drinking Questionnaire, Quantity/Frequency Index, Rutgers Alcohol Problem Index.	1, 3, 6, and 12 months	Both the PFI and PNF groups reported significant reductions in indices of alcohol use relative to control, with participation in any PNF group also associated with significant reductions in alcohol-related negative consequences. PFI and PNF were no different than one another across alcohol use and consequence outcomes. Comparison among PNF conditions supports the use of the “typical student” normative referent.
Lee et al. 2014	Students intending to go on a spring break (SB) trip with friends as well as to engage in heavy episodic drinking (using the 5/4 gender-specific criteria) on at least 1 day of SB (*N* = 783; *N* = 507 who actually went on a SB trip).	Standard BASICS vs. SB-focused BASICS vs. SB-focused BASICS with a friend vs. SB-focused PFI vs. SB-focused PFI with a friend vs. attention control.	Modified Daily Drinking Questionnaire to assess SB drinking intentions (baseline) and actual consumption (followup), 12 items modified from the Young Adult Alcohol Problems Screening Test and the Young Adult Alcohol Consequences Questionnaire to measure anticipated (baseline) and actual (followup) alcohol-related consequences.	1 week after SB	Neither of the PFI conditions (with or without a friend) resulted in reductions in alcohol use or consequences. Only in-person SB-focused BASICS without a friend reduced drinking versus attention control.
Lewis et al. 2014	College students who reported being sexually active within the past year, typically with a member of the opposite sex, and who also reported at least one occasion of heavy episodic drinking in the past month using the 5/4 gender-specific criteria (*N* = 480).	Alcohol-only PNF (PNF-A), alcohol-related risky sexual behavior (RSB) only PNF (PNF-RSB), combined alcohol and alcohol-related RSB PNF (PNF-C), or assessment-only control.	Daily Drinking Questionnaire, Quantity/Frequency Index, Brief Young Adult Alcohol Consequences Questionnaire, additional individual items assessing risky sexual behavior and normative perceptions of sexual behavior adapted from prior work by the first author.	3 and 6 months	Compared with control, PNF-C and PNF-A were associated with reductions in drinking quantity and frequency at 3 months with most effects maintained at 6 months. PNF-C and PNF-RSB were effective in reducing frequency of drinking prior to sex at 3- but not 6-month followup. None of the interventions reduced alcohol-related negative consequences.
Martens et al. 2010	Intercollegiate college athletes (*N* = 263) from three colleges in the Northwest, Midwest, and Northeast.	PFI targeted to college athletes vs. standard PFI targeted to college students in general vs. alcohol education control.	Daily Drinking Questionnaire, Brief Young Adult Alcohol Consequences Questionnaire.	1 and 6 months	Those receiving the targeted PFI who were currently in their athletic season (*N* = 57) or who were heavier drinkers at followup (*N* = 61) reported fewer drinks per week and lower peak BAC, respectively, at 1 month. At 6 months, the effect of the targeted PFI on peak BAC was evident across all participants in that condition, and the standard PFI also showed reductions in peak BAC among heavier drinkers *N* = 57).
Mason et al. 2014	Undergraduates enrolled in psychology courses at a single Southeastern university who scored 8 or higher on the AUDIT (*N* = 18).	Automated personalized text messaging (four to six messages for 4 days that required a brief response) vs. assessment-only control.	AUDIT, additional items assessing quantity and frequency of alcohol use; specific measures used not specified.	1 month	No effects on alcohol use or problems.
Moreira et al. 2012	Freshmen and sophomore college students from 22 universities in the United Kingdom (*N* = 876 at 6 months, 1,050 at 12 months).	E-mailed PNF vs. repeated assessment-only control vs. posttest-only (at 12-month followup) control.	AUDIT, individual items developed by the authors assessing alcohol quantity, frequency and alcohol-related consequences.	6 and 12 months	Compared with repeated-assessment-only control, participants in the PNF group reported less weekly drinking at 6 months (looking at the full sample and a high-risk subsample), but this effect was absent at 12 months. No other effects of the intervention on alcohol use or consequences were evident.
Murphy et al. 2010, study 2	College students reporting at least one occasion of heavy episodic drinking in the past month using the 5/4 gender-specific criteria (*N* = 118).	BASICS vs. e-CheckUpToGo vs. assessment only.	Daily Drinking Questionnaire, individual item assessing number of heavy drinking episodes in the past month.	1 month	Participants assigned to e-CheckUpToGo showed with-in-person reductions in weekly drinking quantity (*d* = 0.42) and frequency of heavy episodic drinking (*d* = 0.39). The e-CheckUpToGo condition was not significantly different than BASICS in terms of reductions in heavy episodic drinking; however, it was also no different than assessment only on this variable or weekly drinking.
Neighbors et al. 2010	Freshmen reporting at least one occasion of heavy episodic drinking in the past month using the 5/4 gender-specific criteria (*N* = 818).	One- vs. four-dose gender-specific PNF vs. one-vs. four-dose gender-neutral PNF vs. attention control.	Daily Drinking Questionnaire, Alcohol Consumption Index, Rutgers Alcohol Problem Index.	6, 12, 18, and 24 months	Biannually administered gender-specific PNF was associated with decreased weekly drinking for men and women, and with fewer-alcohol related consequences for women only. No effects were evident for either of the single-dose PNF conditions or the biannual (four-dose) gender-neutral PNF.
Neighbors et al. 2012	Students intending to engage in heavy episodic drinking (using the 5/4 gender-specific criteria) on their 21st birthday (*N* = 599).	Standard BASICS vs. 21st birthday–focused BASICS vs. 21st birthday–focused BASICS with friend vs. 21st birthday–focused PFI vs. 21st birthday–focused PFI with friend vs. an attention control.	Modified Daily Drinking Questionnaire to measure 21st birthday drinking intentions (baseline) and actual consumption (followup), modified Young Adult Alcohol Problems Screening Test to measure anticipated (baseline) and actual (followup) alcohol-related consequences.	1 week after 21st birthday	21st birthday–focused PFI (without friend) was associated with lower BACs on participants’ 21st birthday compared with control, similar to standard BASICS, but had no effect on total consumption or consequences. 21st birthday–focused PFI with friend reduced alcohol-related consequences relative to control, similar to all three BASICS conditions but did not reduce consumption or BAC.
Palfai et al. 2011	Introductory psychology students reporting two or more occasions of heavy episodic drinking in the past month using the 5/4 gender-specific criteria or who had an AUDIT score of 8 or higher (*N* = 119).	PFI vs. attention control.	Daily Drinking Questionnaire, Young Adult Alcohol Problems Screening Test.	1 month	Those with high (vs. low) levels of alcohol-related consequences at baseline who were assigned to the PFI showed significantly greater reductions in weekly drinking quantity and number of heavy-drinking episodes relative to control participants.
Paschall et al. 2011*a*	Multicampus study (*N* = 30 campuses, 5,074 college freshmen).	AlcoholEdu for College vs. control.	Individual items assessing past-30-day alcohol use, average number of drinks per occasion, and heavy episodic consumption.	N/A (fall and spring assessments were cross-sectional, not longitudinal)	Relative to control campuses, students at colleges assigned to AlcoholEdu for College reported reductions in past 30-day alcohol use and frequency of heavy episodic consumption in the fall; how-ever, these effects were absent at the subsequent spring assessment.
Paschall et al. 2011*b*	Additional findings from Paschall et al. (2011*a*).	See Paschall et al. (2011*a*).	Rutgers Alcohol Problem Index.	See Paschall et al. (2011*a*)	Relative to control campuses, students at colleges assigned to AlcoholEdu for College reported reductions in alcohol consequences in the fall; however, these effects were absent at the subsequent spring assessment.
Patrick et al. 2014	Undergraduates (ages 18–21) who planned to go on a SB trip with their friends (*N* = 263).	Combined SB alcohol use and SB alcohol-related RSB PNF vs. assessment-only control.	Individual items assessing anticipated and actual alcohol use, sexual behavior, and associated consequences.	1 week after SB	No significant differences between PNF and control on alcohol use, risky sexual behavior or related consequences.
Schuckit et al. 2012	Freshmen who have never met criteria for DSM–IV alcohol or drug dependence, who reported any drinking in the past 6 months and who reported a low or high subjective level of response (LR) to alcohol (*N* = 64).	Prevention videos tailored to a low LR to alcohol vs. non-tailored prevention videos.	Individual items assessing alcohol use and associated consequences (drawn from the Rutgers Alcohol Problem Index).	Immediate posttest and 4 weeks following end of the intervention	Although all participants showed significant decreases in typical and peak drinks per occasion, participants with a low LR who were assigned to the tailored group showed greater reductions than those assigned to the nontailored group. Additionally, in terms of typical drinks per occasion, those with high LR assigned to the nontailored group showed greater reductions than those in the tailored group.

## Footnotes

1 Wechsler and colleagues (1995) define an occasion of heavy episodic consumption as five or more drinks for men and four or more drinks for women in a row. This definition was used most frequently across the studies reviewed here; although, the statistics from the Monitoring the Future study (Johnston et al. 2013) do not differentiate by gender, and only indicate the percentage of young adults and college students (both men and women) who consumed five or more drinks on a single occasion.

2 The AUDIT-C is a three-item alcohol screening test that is scored on a scale from 0 to 12.
